# Exploring the Renoprotective Potential of Bioactive Nutraceuticals in Chronic Kidney Disease Progression: A Narrative Review

**DOI:** 10.7759/cureus.68730

**Published:** 2024-09-05

**Authors:** Anindita Ghosh, Arti Muley, Sakshi Bhat, Archana Ainapure

**Affiliations:** 1 Nutritional Sciences and Dietetics, Symbiosis Skills and Professional University, Pune, IND; 2 Nutrition and Dietetics, Symbiosis School of Culinary Arts, Pune, IND; 3 Nutrition and Dietetics, Symbiosis International (Deemed University), Pune, IND; 4 Nutrition, Texas School of Mental Health, Pune, IND

**Keywords:** bioactive, chronic kidney disease, glomerular filtration rate, nutraceuticals, oxidative stress, proteinuria

## Abstract

Chronic kidney disease (CKD) is a condition that is characterized by progressive loss of kidney function over time. A substantial increase in the burden of CKD is evident globally, attributed to multifactorial conditions like an expanding aging population, rising diabetes and hypertension rates, and more significant exposures to risk factors associated with the environment and lifestyle. Nutraceuticals are substances that are usually considered a food or an active part of a food that provides medical or health benefits, including the prevention and treatment of a disease. The aim is to review the positive role of nutraceuticals in managing CKD. A narrative review is generated, extracting the papers from databases like Web of Science, Scopus, ScienceDirect, ResearchGate, and PubMed. Animal and human trials focusing on the effect of different nutraceuticals on the initial stage of kidney disease, i.e., stages 1, 2, and 3 of CKD, were included. The review's outcome is understanding the effectiveness of nutraceuticals that have shown positive results in CKD conditions. Active compounds include ubiquinone, curcumin, nitrates, nitrites, lycopene, and resveratrol. These bioactive components are also beneficial for other comorbid conditions like diabetes, hypertension, and cardiovascular conditions that have an eminent adverse effect on CKD. Lycopene, coenzyme Q10 (CoQ10), resveratrol, curcumin, and flavonoids have positively impacted CKD complications. Nutraceuticals hold great promise for individuals with CKD in the coming years, offering diverse potential benefits. These include delivering vital antioxidant and anti-inflammatory support to alleviate oxidative stress and inflammation, helping to regulate blood pressure and lipid levels for improved cardiovascular health, promoting optimal renal function to sustain kidney health, assisting in maintaining electrolyte balance, warding off complications, influencing gut microbiota for enhanced digestive well-being, and ultimately elevating the overall quality of life, for those managing CKD.

## Introduction and background

Deterioration of renal function over time leading to end-stage renal disease (ESRD) is the hallmark of chronic kidney disease (CKD), a primary global health concern [1]. Globally, the prevalence of CKD is on the rise due to factors such as aging populations, diabetes, and hypertension. Over 10% of people worldwide suffer from CKD, according to a 2024 update by The Lancet Global Health [2]. Around 13.4% (11.7-15.1%) is the estimated prevalence of CKD worldwide, and its prevalence is increasing worldwide because of the increased rate of many types of lifestyle diseases [3]. According to the National Kidney Foundation (NKF), CKD is defined as impaired kidney structure and functioning caused by various types of comorbid conditions and lifestyle complications with an unexplained glomerular filtration rate (GFR) of less than 60 mL/min per 1.73 m^2^ for more than three months. The pathophysiology of CKD involves a progressive loss of glomerular filtration capacity, which intensifies oxidative stress and systemic inflammation and accelerates the disease's development course [4]. Diagnosis of CKD is a challenge, as unless the estimated glomerular filtration rate (eGFR) drops to 30 mL/min per 1.73 m^2^, CKD might be subtle and cause no symptoms for months or even decades [5]. According to a study published in the Lancet, 1.2 million people worldwide passed away from CKD in 2017 [6]. Renal experts projected that by 2040, CKD would rank as the sixth most common cause of mortality globally [7]. Innovative therapeutic options are urgently needed since CKD continues to be a significant source of morbidity and mortality despite advances in medical therapy [1].

In recent years, growing attention has been given to the possible renoprotective benefits of bioactive nutraceuticals. The phrase "nutraceutical" is a combination of nutrition and pharmaceuticals. According to reports, DeFelice and the Foundation for Innovation in Medicine came up with the term in 1989. In 1994, a press release restated and refined its definition, which was defined as "any substance that provides medical or health benefits, including the prevention and treatment of disease, and may be considered a food or part of a food." These products could be anything from diet plans, supplements, and separated nutrients to processed foods, herbal goods, genetically modified "designer" foods, and genetically altered foods [8]. Nutraceuticals contain specific bioactive food components, which come from natural sources like plants and marine life and provide a variety of physiological characteristics that go beyond simple dietary needs. Remarkably, their anti-inflammatory, antioxidant, and metabolic regulatory qualities point to their potential therapeutic uses in the treatment of CKD [9]. Several nutraceuticals have shown promising effects on CKD, such as reducing inflammation, oxidative stress, and cellular fibrosis, regulating intestinal microbiota modulation and immunomodulation, and improving renal function and cardiovascular outcomes. Anti-inflammatory agents like curcumin can contribute to reducing systemic inflammation linked to CKD, while antioxidants like vitamin E and omega-3 fatty acids have been found to lower oxidative stress [10].

A growing paradigm in personalized and integrative medicine is being supported by the exploration of bioactive nutraceuticals as supplemental or alternative therapies for CKD. Nutraceuticals are potentially adjunctive therapies in CKD management, but further research is necessary to determine their optimal dosages, formulations, and long-term effects [9]. They should be thoroughly investigated through rigorous scientific research and clinical trials due to their potential to attenuate renal injury, decrease disease progression, and improve outcomes in individuals with CKD.

This review aims to compile the body of knowledge regarding the bioactive components as nutraceuticals linked to renal protection and their mechanisms of action in the management of CKD and to explore their therapeutic impact on reducing the progression of CKD.

## Review

Methodology

The proposed review used a comprehensive search strategy by implementing database searches from PubMed, ScienceDirect, Web of Science, Scopus, and ResearchGate for the information source using Medical Subject Headings (MeSH) terms related to nutraceuticals and CKD. The search terms included "chronic kidney disease," "CKD," "nutraceuticals," "bioactive components," "ubiquinone," "nitrates," "nitrites," "quercetin," "resveratrol," "lycopene," "coenzyme Q10," "curcumin," and "flavonoids." Boolean operators (AND, OR) were used to combine each term. The eligibility criteria of the searches depended on the inclusion and exclusion criteria of the review.

Inclusion criteria were studies and trials on animal and human participants examining the effect of specified nutraceuticals (ubiquinone, nitrates, nitrites, quercetin, lycopene, coenzyme Q10 (CoQ10), curcumin, resveratrol, and flavonoids) on managing the initial stages (stages 1, 2, and 3) of CKD between 2000 and 2024. Both human and animal studies were included, as including both kinds of investigations ensured detailed and complete evidence on the effect of nutraceuticals on CKD. When paired with human trials, animal research can shed light on nutraceuticals' early-stage effects and possible side effects, giving a complete picture of their safety and effectiveness. The selection of the following nutraceuticals was made since the majority of these physiologically active ingredients, ubiquinone, quercetin, lycopene, CoQ10, curcumin, resveratrol, and flavonoid, have shown significant results in the past in clinical domains. By evaluating their efficacy and safety in practical applications, their inclusion offers insightful information for clinical practice.

The exclusions were conference papers, editorials, letters, and studies without any primary data; studies focusing on stages 4 and 5 of CKD; and studies not involving specified nutraceuticals beyond the time period of 2000-2024. The search led to a total of 462 papers. After the initial search, duplicates were removed manually, and the remaining records, i.e., 356, were screened on the basis of the abstracts and titles. Nineteen eligible studies comprising 10 clinical trials and nine systematic reviews and meta-analyses were recovered after assessing each abstract and title for establishing relevance to the inclusion and exclusion criteria. 

Results

Nutraceuticals are popular in CKD management since they reduce the progression of the disease stage and improve the quality of life and the symptoms associated with the disease. Numerous nutraceuticals, including polyphenols, curcumin, and omega-3 fatty acids, have anti-inflammatory and antioxidant qualities. Oxidative stress and inflammation are prevalent in CKD and aid in the course of the illness [10]. Nutraceuticals can help prevent kidney injury and preserve improved renal function by lowering these risk factors. Probiotics and prebiotics can enhance gut health and lower uremic toxins, which build up as renal function deteriorates [11]. This improves quality of life by relieving symptoms like weariness and nausea while also supporting kidney function [12]. CoQ10 and vitamin D are instances of nutraceuticals that help reduce hypertension, diabetes, and cardiovascular complications, comorbidities that are common in CKD [10]. Appropriate medical care for these illnesses lessens the burden on the kidneys and helps to impede the advancement of the illness. Symptoms of CKD include weariness, itching, cramping in the muscles, and difficulty sleeping [3]. Vitamin B complex, magnesium, and several herbal supplements are nutraceuticals that can reduce symptoms and improve a patient's quality of life [5]. Nutraceuticals provide a supplementary strategy to conventional CKD care, addressing the advancement of the illness and enhancing patient well-being. CoQ10, flavonoids, epigallocatechin-3-gallate (EGCG), tannic acid, curcumin, and resveratrol are some of the most well-known bioactive compounds, among many others, that can manage the progression of the disease [12]. Out of 19 studies comprising randomized clinical trials (RCTs), systematic reviews, and meta-analyses, 10 RCTs, both double-blind placebo-controlled and non-double-blind placebo-controlled, were included which were conducted on nutraceuticals primarily consisting of the bioactive components CoQ10 (ubiquinol), flavonoids, curcumin, and polyphenols in CKD patients to identify the renoprotective benefits of these bioactive components on the initial stages of CKD without displaying any kind of side effects in the participants. 

CoQ10

CoQ10 is a group of nutraceuticals involved in producing ATP, regarded as the primary and most efficient energy source for cellular functioning and performance in the body. CoQ10 has the potential to enhance renal function, reduce proteinuria, diminish levels of oxidative stress indicators malondialdehyde (MDA) and superoxide dismutase (SOD), and shield the heart from cardiovascular complications [13]. Ubiquinone is the oxidized form, while ubiquinol is the reduced form. Its administration improves inflammation levels, glucose metabolism, cardiac structure, and cardiac biomarkers. Ubiquinol has more excellent antioxidant activity than ubiquinone, making it potentially more beneficial in conditions associated with oxidative stress, such as CKD. In an RCT conducted by Ishikawa et al., 600 mg/kg body weight/day of ubiquinol was trialed orally in 10 male three-week-old heminephrectomized Sprague-Dawley rat on high salt (0.8%) for four weeks. Significant reduction in systolic blood pressure, urinary albumin excretion, and superoxide anion generation was reported, improving the renal function in the trialed rats [14]. Oxidative stress contributes to the progression of CKD, and studies suggest that ubiquinol intake may help reduce oxidative stress and improve renal function in individuals with CKD [15]. CoQ10 is a crucial nutrient in the mitochondrial electron transport chain, where it plays a vital role in energy production. CoQ10 enhances mitochondrial function, improving cellular energy production and protecting against oxidative damage to renal cells [16]. In a double-blind randomized placebo-controlled study conducted in 2017 by Gholnari et al. on 50 participants with diabetic nephropathy, CoQ10 supplementation 100 mg/day for 12 weeks caused a decrement in serum insulin levels, homeostasis model of assessment-estimated insulin resistance, homeostasis model of assessment-estimated B-cell function, plasma malondialdehyde (MDA), and advanced glycation end product (AGE) levels promoting endothelial health by improving endothelial function, enhancing nitric oxide production, and reducing oxidative stress in blood vessels, supporting proper renal blood flow, vascular health, and kidney function [17,18]. CoQ10 has shown modest antihypertensive effects, reducing oxidative stress and modulating vascular tone. By helping to control blood pressure, CoQ10 may contribute to managing CKD and reducing the risk of cardiovascular complications [19]. No adverse effects related to CoQ10 administration were reported in the trial conducted.

Flavonoids

Flavonoids are an active group of natural compounds widely distributed in various plant-based foods, such as fruits, vegetables, and herbs. They have been recognized for their potential health benefits, including anti-inflammatory, antioxidant, and anti-fibrotic properties. In recent years, increasing interest has been in exploring the potential therapeutic effects of flavonoids in CKD. Flavonoids reduce oxidative stress, inflammation, and fibrosis, improving endothelial function and modulating immune responses [20]. In an RCT by Guo et al. in 2015 on age-matched eight-week-old female MRL/lpr mice, astilbin, a flavonoid in its natural form, isolated from the rhizome of *Smilax glabra* was administered at 10, 20, or 40 mg/kg, respectively, in eight, 14, and eight mice groups till the 30th week. A reduction of glomerular size at the 30th week with 20 mg/kg and 40 mg/kg astilbin showed lowered inflammation in systemic lupus erythematous-induced glomerulonephritis [21]. Quercetin, epicatechin, curcumin, resveratrol, and genistein show promising results in experimental and preclinical studies [22]. These flavonoids have demonstrated potential renoprotective effects by targeting multiple pathways involved in CKD progression [23]. It suggests flavonoids may have renoprotective effects and could be considered a complementary approach to managing CKD. However, more research is needed to fully understand their mechanisms of action and validate their clinical effectiveness [24]. In an RCT by Wang et al., in 2015, 21-one-week-old hypertensive rats were supplemented with grape seed extract containing proanthocyanidin 100-250 mg/kg/day for 22 weeks which reduced their proteinuria and improved the systolic blood pressure enhancing renoprotection [25].

EGCG

EGCG is a primary natural polyphenolic compound in green tea, known for its antioxidant, anti-inflammatory, and anti-cancer properties. EGCG has a promising effect as a protective agent against kidney diseases. Its antioxidant, anti-inflammatory, and anti-fibrotic properties make it a valuable addition to the treatment strategies for various kidney conditions. The protective effects of EGCG in different kidney diseases, including diabetic nephropathy, drug-induced kidney injury, renal fibrosis, and kidney stone formation, make EGCG a prominent bioactive compound in kidney disease management. Its antioxidant properties help to reduce oxidative stress and prevent cellular damage [26]. Peng et al. in the year of 2011 conducted an RCT on crescentic glomerulonephritis induced in 129/svJ mice where administration of 50 mg/kg body weight/day of EGCG was done for three weeks. A renoprotective action was observed on a lower dose of EGCG administration of 25 mg/kg body weight/day for two weeks evident by a decrease in proteinuria and serum creatinine levels [27]. The anti-inflammatory effects of EGCG inhibit the production of pro-inflammatory molecules and reduce immune cell infiltration [28]. EGCG is reported to have anti-fibrotic effects, inhibiting the activation of fibroblasts and the deposition of extracellular matrix proteins that contribute to renal fibrosis [29]. EGCG can potentially prevent kidney stone formation by modulating crystal growth and inhibiting crystal adherence to renal epithelial cells [30].

Tannic Acid

The potential role of tannic acid in improving renal function recovery following renal warm ischemia-reperfusion injury is observed in a rat model. Renal warm ischemia-reperfusion injury occurs when blood flow to the kidneys is temporarily interrupted and restored. It is a common cause of acute kidney injury and can lead to long-term renal dysfunction [31]. The study involved inducing renal warm ischemia-reperfusion injury in rats and treating them with tannic acid. Various parameters related to renal function, oxidative stress, inflammation, and tissue damage were evaluated in these rat models. The results demonstrated that treatment with tannic acid significantly improved renal function recovery in the rat model [32]. Tannic acid administration reduced creatinine and blood urea nitrogen (BUN) levels, the active indicators of kidney function [33]. Tannic acid treatment reduced oxidative stress markers and inflammatory mediators in the kidney tissues, indicating its antioxidant and anti-inflammatory effects. The authors also observed that tannic acid treatment reduced tissue damage and apoptosis (cell death) in the kidneys. These findings suggest that tannic acid may protect renal tissues during ischemia-reperfusion injury [34]. Overall, the study suggests that tannic acid has the potential to improve renal function recovery and protect against renal injury in the context of warm ischemia-reperfusion.

Lycopene

Lycopene is a natural pigment and a powerful antioxidant found in various fruits and vegetables, with tomatoes being one of the primary sources. It has been widely studied for its potential health benefits, particularly its role in reducing the risk of chronic diseases [35]. Lycopene in other components like carotene, tocopherol, retinol, zeaxanthin, and leptin is a protective factor concerning CKD in human and animal studies. Various other types of pathways have been shown to reduce the progression of CKD [36]. El-Gerbed conducted an RCT in 2014 on 10 albino rats with deltamethrin (insecticide)-induced nephrotoxicity. On administration of 1 mg/kg/day of lycopene in these rats for 30 days, there was a lower nephrotoxic effect documented in the rats [37]. Another similar double-blind placebo-controlled RCT was conducted by Pierine et al. in 2014 on 14 obese male Wistar rats. The trialed rats were on a high-fat diet and sucrose for six weeks and received a supplementation of lycopene 10 mg/kg body weight mixed with corn oil for five days per week orally every morning. After six weeks of lycopene administration, these rats showed an improvement in the receptors of AGEs and tumor necrosis factor-alpha (TNF-α) reducing oxidative stress and inflammation [38]. CKD patients may have lower levels of retinol due to impaired kidney function. Adequate vitamin A intake is essential for maintaining immune function and vision [39]. Zeaxanthin and lutein are carotenoids primarily known for their role in eye health and vision and CKD patients; they have also been shown to reduce mortality [40]. Lycopene is believed to protect against oxidative stress-induced renal injury and nephrotoxicity by neutralizing free radicals and reducing inflammation. Lycopene's effects are also prominent in reducing kidney-related conditions, such as renal fibrosis, diabetic nephropathy, kidney stone formation, and drug-induced kidney injury. These studies suggest that lycopene supplementation may have beneficial effects in mitigating kidney damage and improving renal function [41]. Lycopene can modulate gene expression, inhibit inflammatory pathways, and regulate oxidative stress in renal tissue [42]. Lycopene has been reported to inhibit the crystallization of calcium oxalate, a principal constituent of kidney stones. By reducing oxalate production and modulating urinary pH, lycopene may play a crucial role in preventing the formation and growth of kidney stones [43].

Fabaceae, Cranberry, and Dandelion

Effects of a nutraceutical diet containing *Lespedeza* spp., *Vaccinium macrocarpon* (cranberry), and *Taraxacum officinale* (dandelion) on spontaneous CKD in *Felis catus* are noted in an animal study. CKD ranges from mild to severe conditions in older *Felis catus* and is characterized by a gradual loss of kidney function. The study involved feeding cats with a diet supplemented with *Lespedeza *spp., cranberry, and dandelion. Various parameters related to kidney function, including BUN, creatinine, and proteinuria, were assessed. Evaluation of markers of oxidative stress and inflammation was also done. The results showed that the nutraceutical diet improved several critical aspects of feline CKD. Cats fed the supplemented diet demonstrated reduced BUN and creatinine levels, indicating improved kidney function. Proteinuria, the presence of excess protein in the urine, was decreased in cats on the nutraceutical diet. Reduction of oxidative stress markers and pro-inflammatory cytokines in cats fed the nutraceutical diet was also observed. The study suggests that a nutraceutical diet based on *Lespedeza *spp., cranberry, and dandelion can potentially improve spontaneous feline CKD [44]. The diet's beneficial effects on kidney function, reduction of proteinuria, and attenuation of oxidative stress and inflammation are promising.

Curcumin

Curcuminoids, including curcumin, demethoxycurcumin, and bisdemethoxycurcumin, are the active compounds found in turmeric. Khajehdehi et al. in 2011 conducted a double-blind placebo-controlled RCT on 40 type 2 diabetic nephropathy patients (20 cases and 20 controls) where three capsules per day containing 500 mg turmeric with bioactive component 22.1 mg curcumin were supplemented in the trial group and the same quantity of starch capsules was given in placebo group for two months. The authors mentioned a decrement in the serum levels of tumor growth factor-beta (TGF-β), interleukin levels (IL-8), and proteinuria post-supplementation [45]. Another similar placebo-controlled RCT was conducted by the same group of researchers in 2012 on 24 lupus nephritis patients (12 trials and 12 controls) where the trial patients received 500 mg of turmeric supplementation in capsular form containing 22.1 mg of bioactive curcumin, one capsule thrice daily orally for three months. The controls received the same number of capsules containing the same quantity of starch for a similar duration. There was a significant decrease in proteinuria, hematuria, and systolic blood pressure among the trialed patients which provided a renoprotective effect [46]. Curcuminoids have relatively poor bioavailability, and thus, various formulations and delivery methods, such as combining curcuminoids with black pepper extract (piperine) or using nanoparticle technology, are being explored to enhance their absorption and bioavailability [47]. Curcumin's potential mode of action in CKD is by focusing on its effects on endogenous intestinal alkaline phosphatase (IAP) [48]. IAP is an enzyme found in the intestines that plays a crucial role in maintaining gut homeostasis and protecting against inflammation and oxidative stress [49]. Curcumin stimulates the production and activity of IAP, leading to beneficial effects in CKD. The activation of IAP by curcumin is suggested to have multiple protective mechanisms, including the reduction of oxidative stress, inflammation, and fibrosis in the kidneys. By enhancing IAP activity, curcumin may help maintain gut barrier integrity and reduce the translocation of toxins and harmful substances into the bloodstream, positively impacting kidney health in CKD patients [50].

Resveratrol

Resveratrol is a balanced and natural polyphenolic compound with various subtypes found in various plants, including grapes and berries; it has gained attention for its antioxidant, anti-inflammatory, and anti-aging properties [51]. Resveratrol exhibits strong antioxidant properties, which can counteract oxidative stress and significantly contribute to CKD progression. By limiting the excessive use of the reactive oxygen species (ROS) compound, a ROS, resveratrol, may help protect the kidneys from oxidative damage [52]. In a double-blind placebo-controlled RCT by Ghanim et al., in 2010, 10 normal healthy individuals were administered *Polygonum cuspidatum* extract (PCE) containing 40 mg resveratrol per day for six weeks which lowered their plasma TNF-α, IL-6, and C-reactive protein (CRP) concentrations displaying a renoprotective activity in the healthy individuals [53]. Stilbenes are a group of polyphenol compounds that include resveratrol. In addition to resveratrol, other stilbenes, such as pterostilbene and piceatannol, are also being investigated for their potential health benefits. Similar to resveratrol, stilbenes exhibit antioxidant and anti-inflammatory properties, which have protective effects on the kidneys. Chronic inflammation is a common feature of CKD. Resveratrol has been shown to possess anti-inflammatory properties by inhibiting the activation of inflammatory pathways and reducing the production of pro-inflammatory molecules. This modulation of inflammation may help slow the progression of CKD and alleviate related complications [54]. Resveratrol has been found to benefit kidney function. It can improve endothelial function, promote vasodilation, and enhance renal blood flow. These effects contribute to the protection of renal tissues and the preservation of kidney function [55]. Resveratrol activates a group of enzymes called sirtuins, which play a role in various cellular processes, including DNA repair, energy metabolism, and stress response. Activation of sirtuins by resveratrol may positively impact kidney health and CKD progression [56].

Table 1 displays the nutraceutical effects of CoQ10, ubiquinone, ubiquinol, lycopene, curcumin, resveratrol, and flavonoids on reducing the progression of CKD.

**Table 1:** Effect of specific nutraceutical interventions with dosages and duration on chronic kidney disease: coenzyme Q10, lycopene, curcumin, resveratrol, and flavonoids DN: diabetic nephropathy; MDA: malondialdehyde; AGE: advanced glycation end product; EGCG: epigallocatechin-3-gallate; PCE: *Polygonum cuspidatum* extract

Author details	Type of study	Participants	Intervention	Dosage	Duration	Outcome	Adverse effects of the nutraceuticals administered
Gholnari et al., 2018 [17]	Double-blind randomized placebo-controlled clinical trial	50 participants with DN	Coenzyme Q10 supplements	100 mg/day	12 weeks	Decreases in serum insulin levels, homeostasis model of assessment-estimated insulin resistance, homeostasis model of assessment-estimated B-cell function, plasma MDA, and AGE levels	None reported
Khajehdehi et al., 2011 [45]	Double-blind randomized placebo-controlled clinical trial	40 patients with overt type 2 DN were randomized into a trial group (n=20) and a control group (n=20)	22.1 mg curcumin in the trial group and starch capsules in the placebo group	3 capsules/day, 1 capsule containing 500 mg turmeric (22.1 mg curcumin)	2 months	Decreased serum levels of tumor growth factor and interleukin-8, urinary protein excretion post-supplementation	None reported
Pierine et al., 2014 [38]	Double-blind randomized placebo-controlled clinical trial	14 obese male Wistar rats	Rats were fed on a high-fat diet and sucrose. Trial rats received lycopene mixed with corn oil, and the placebo group received plain maize oil	10 mg/kg body weight lycopene 5 days per week orally every morning	6 weeks	Improvement in receptors of AGEs and tumor necrosis factor-alpha reducing oxidative stress and inflammation	None reported
Guo et al., 2015 [21]	Randomized clinical trial	Age-matched female MRL/lpr mice	Astilbin, a flavonoid in its natural form, isolated from the rhizome of *Smilax glabra* administration in eight-week-old MRL/lpr mice 10, 20, or 40 mg/kg, respectively, in eight, 14, and eight mice groups	Initial oral administration of astilbin in 0.5% methylcellulose on alternate days	30th week	Reduction of glomerular size at 30 weeks with 20 mg/kg and 40 mg/kg astilbin showing lowered inflammation in systemic lupus erythematous-induced glomerulonephritis	None reported
Peng et al., 2011 [27]	Randomized clinical trial	Crescentic glomerulonephritis induced in 129/svJ mice	EGCG	50 mg/kg body weight/day	3 weeks	A lower dose of EGCG (25 mg/kg body weight/day for two weeks) treatment decreased proteinuria and serum creatinine and markedly improved renal histology	None reported
El-Gerbed, 2014 [37]	Randomized clinical trial	10 albino rats with deltamethrin (insecticide)-induced nephrotoxicity	Lycopene	1 mg/kg/day	30 days	Renoprotective action of lycopene in the nephrotoxic condition of lycopene administration	None reported
Ghanim et al., 2010 [53]	Double-blind, placebo-controlled randomized clinical trial	10 normal weight healthy individual	PCE containing resveratrol	40 mg/day	6 weeks	Lower plasma concentrations of tumor necrosis factor-alpha, interleukin-6, and C-reactive protein	None reported
Khajehdehi et al., 2012 [46]	Placebo-controlled randomized clinical trial	24 lupus nephritis patients (12 cases; 12 controls)	Trial patients (n=12) received turmeric in capsules. Control patients (n=12) received starch in similar quantity and duration	1 capsule thrice daily of 500 mg turmeric containing 22.1 mg bioactive curcumin in trial patients	3 months orally	Decrease in proteinuria, hematuria, systolic blood pressure	None reported
Wang et al., 2015 [25]	Randomized clinical trial	21-week-old hypertensive rats	Grape seed proanthocyanidin extract	100-250 mg/kg/day	22 weeks	Reduced proteinuria and systolic blood pressure	None reported
Ishikawa et al., 2011 [14]	Randomized clinical trial	10 heminephrectomized male three-week-old Sprague-Dawley rat on high salt (0.8%)	Ubiquinol, a reduced form of coenzyme Q10	600 mg/kg body weight/day	4 weeks	Reduced systolic blood pressure, urinary albumin excretion, and superoxide anion generation, improving renal function	None reported

Discussion

With its rising prevalence and significant effects on morbidity and mortality, CKD is a major global public health concern. The progressive nature of CKD makes it necessary to investigate alternative interventions in addition to conventional therapies to enhance current treatment approaches [57]. In the context of CKD, nutraceuticals, which are defined as bioactive compounds derived from food sources with potential therapeutic benefits, emerged as a promising field of study [58]. The literature on the use of nutraceuticals to slow the progression of CKD is critically reviewed in this discussion, which also covers the clinical implications of implementing these interventions into CKD treatment and possible mechanisms for their effects.

Nutraceuticals' Antioxidant and Anti-inflammatory Properties

Nutraceuticals' anti-inflammatory and antioxidant characteristics are two main ways in which they may slow the course of CKD. In CKD, oxidative stress and inflammation play major roles in renal damage. Several nutraceuticals have shown promise in reducing these processes [59]. The antioxidant properties of polyphenols, which are present in a wide range of fruits, vegetables, and plant-based diets, have been well-researched. Grapes contain a polyphenol called resveratrol, which has been demonstrated to modulate oxidative stress pathways and attenuate inflammation. This may lessen the strain on the kidneys and slow the progression of CKD [60-62]. The anti-inflammatory properties of omega-3 fatty acids, which are found in large amounts in fish oil, may also affect the inflammatory pathways connected to CKD. According to experimental research, omega-3 fatty acids may lessen renal fibrosis and proteinuria, two indicators of the advancement of CKD. However, more research using carefully thought-out clinical trials with reliable methodologies is needed to translate these findings into clinical practice [63]. Bioactive substances that inhibit pro-inflammatory mediators and signaling pathways, such as quercetin, which is found in large quantities in onions and apples, and curcumin, which is derived from turmeric, have been shown to have anti-inflammatory properties. These natural compounds may play a role in slowing the progression of CKD, as experimental studies have demonstrated encouraging results in reducing renal inflammation and fibrosis. Clinical trials are necessary to confirm these results and assess their effectiveness in human participants [64].

Adjustment of Renal Hemodynamics and Function

Natural bioactive substances may improve renal hemodynamics and function, slowing the progression of CKD [65]. For instance, in animal models of CKD, flavonoids from citrus fruits and berries have been demonstrated to improve renal blood flow and GFR. Enhanced GFR results from the combination of the multifactorial processes comprising anti-inflammatory, antioxidant, vasodilatory, podocyte-protective, and anti-fibrotic effects leading to an improvement in the renal glomeruli. These nutraceuticals may lessen the loss of kidney function linked to the advancement of CKD by enhancing renal perfusion and function [61]. Furthermore, some bioactive ingredients have diuretic qualities that help with sodium excretion and fluid retention, both of which help control hypertension and fluid overload associated with CKD [62]. Moreover, the potential renoprotective effects of bioactive peptides derived from food proteins, like soy and whey protein, have attracted attention. These peptides can inhibit the angiotensin-converting enzyme (ACE), which lowers blood pressure and causes vasodilation. They might also alter the pathways leading to renal fibrosis and inflammation, which would help to maintain renal function [66]. Clinical trials using dietary supplements of bioactive peptides have shown improvements in GFR and proteinuria, underscoring their potential as adjuvant therapies in managing CKD [67].

Management of Renal and Metabolic Health

An essential part of managing CKD is promoting renal health and controlling metabolic parameters, both of which are facilitated by natural bioactive compounds [68]. Omega-3 fatty acids from fish oil can enhance insulin sensitivity and lipid profiles, which lowers cardiovascular risk factors in individuals with CKD. Additionally, these fatty acids have anti-inflammatory qualities that could guard against kidney damage and delay the advancement of CKD. Nitric oxide helps in maintaining kidney health. NO3− and NO2− are dietary sources of nitric oxide. Certain vegetables, such as spinach and beetroot, are rich in these compounds. Studies suggest that dietary nitrate improves blood pressure control, endothelial function, and exercise tolerance in CKD patients [69]. Furthermore, bioactive substances like anthocyanins, present in red grapes and berries, have been linked to insulin sensitivity and glucose metabolism control. Anthocyanins may help manage CKD-related complications in diabetic CKD models by enhancing glycemic control and lowering oxidative stress, according to experimental research. However, more investigation is needed [70].

Challenges and Prospects for the Future

Several obstacles and knowledge gaps persist despite the encouraging data indicating the potential of natural bioactive components as nutraceuticals to manage CKD. Many studies are preclinical or small-scale clinical trials, which restricts the applicability of their conclusions in real-world contexts. Furthermore, there are significant differences in the bioavailability and metabolism of bioactive components, which can impact their clinical outcomes and therapeutic efficacy. Further research is necessary to determine the natural bioactive component supplementation's ideal dosage, composition, and duration. A personalized approach to managing CKD is imperative due to the potential for medication interactions and individual variability in response to nutraceutical interventions. Additionally, patients face practical difficulties in adhering to dietary guidelines and making lifestyle changes, underscoring the significance of patient education and assistance in implementing nutraceuticals.

**Figure 1:**
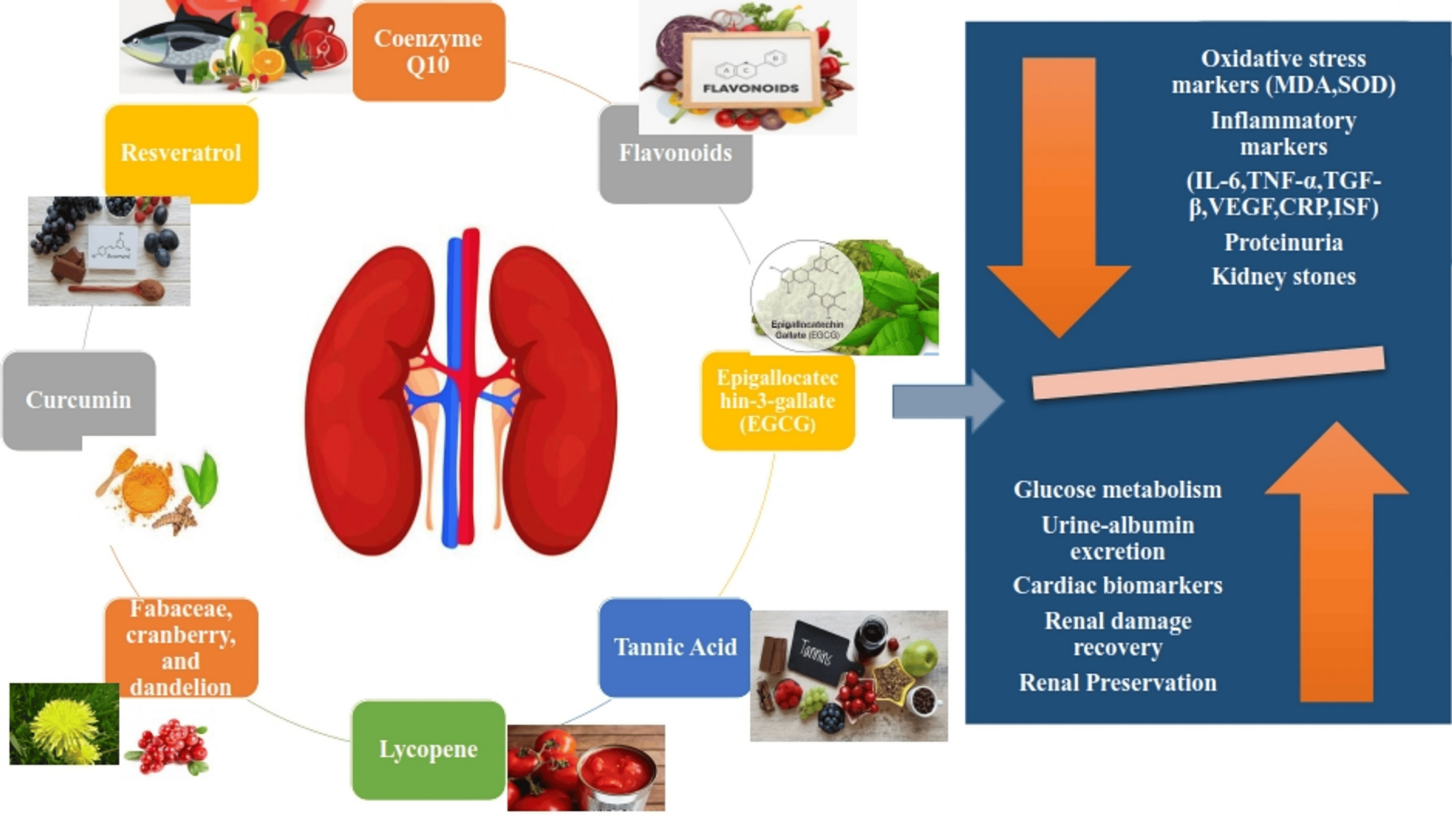
Interplay of different bioactive components in the reduction of oxidative stress biomarkers (MDA, SOD), inflammatory biomarkers (IL-6, TNF-α, TGF-β, VEGF, CRP, ISF), proteinuria, and kidney stones and improvement of glucose metabolism, urine-albumin excretion, cardiac biomarkers, renal damage recovery, and renal preservation MDA: malondialdehyde; SOD: superoxide dismutase; IL-6: interleukin-6; TNF-α: tumor necrosis factor-alpha; TGF-β: transforming growth factor-beta; VEGF: vascular endothelial growth factor; CRP: C-reactive protein; ISF: insulin sensitivity factor Image Credit: Anindita Ghosh

Figure 1 shows the interplay of different bioactive components.

## Conclusions

Supplementation of bioactive compounds such as CoQ10, lycopene, flavonoids, curcumins, and polyphenols in the form of nutraceuticals in specific dosages and for a specific duration poses crucial importance in repressing systemic responses by lowering inflammatory processes, oxidative damage, and receptive overdrive; increasing the rate of glomerular filtration and kidney-specific blood circulation; stimulating antioxidant attributes; enhancing nitric oxide availability; preventing interstitial renal tissue scarring; and promoting tubular epithelial regeneration. The renoprotective action of these nutraceuticals in CKD deserves special mention, as, along with their functionality, they are not contributors to any adverse effects. Evident research on the effects of nutraceuticals on CKD in animal subjects and in vitro suggests that these supplements can slow the progression of renal damage and prevent ESRD in the long run. An effective way of implementing nutraceuticals as an adjunct and renoprotective therapy to manage CKD is to provide them in definite dosages, combinations, and durations along with proper dietary and lifestyle interventions.
